# A Healthful Pull

**Published:** 1855-03

**Authors:** 


A Healthful Pull.—Many a man knows how much it contributes to his health and happiness to find peace, and quiet, and unity at home. *An illustration*,—"A bridegroom requested his wife to accompany him into the garden a day or two after the wedding. He then threw a line over the roof of their cottage. Giving her one end of it, he retreated to the other side, and exclaimed,—'Pull the line!' She pulled at his request as far as she could. He cried 'Pull it over.' 'I can't!' she replied. 'Pull with all your might,' shouted the whimsical husband. But in vain were all the efforts of the bride to pull over the line, so long as the husband held on the end. But when he came round, and they both pulled at one end, it came over with great ease. 'There,' said he, 'you see how hard and ineffectual was our labor, when we pulled in opposition to each other, but how easy and pleasant it is when we pull together. If we oppose each other, it will be hard work; if we act together it will be pleasant to live. Let us, therefore, always pull together.'"

